# Human Papillomavirus 16 E5 Modulates the Expression of Host MicroRNAs

**DOI:** 10.1371/journal.pone.0021646

**Published:** 2011-07-01

**Authors:** Dario Greco, Niina Kivi, Kui Qian, Suvi-Katri Leivonen, Petri Auvinen, Eeva Auvinen

**Affiliations:** 1 DNA Sequencing and Genomics Laboratory, Institute of Biotechnology, University of Helsinki, Helsinki, Finland; 2 Department of Bioscience and Nutrition, Karolinska Institutet, Stockholm, Sweden; 3 Department of Virology, Haartman Institute, University of Helsinki, Helsinki, Finland; 4 Medical Biotechnology, VTT Technical Research Centre of Finland, Turku, Finland; 5 Department of Virology and Immunology, Helsinki University Hospital Laboratory, Helsinki, Finland; Karolinska Institutet, Sweden

## Abstract

Human papillomavirus (HPV) infection is a prerequisite of developing cervical cancer, approximately half of which are associated with HPV type 16. HPV 16 encodes three oncogenes, E5, E6, and E7, of which E5 is the least studied so far. Its roles in regulating replication and pathogenesis of HPV are not fully understood. Here we utilize high-throughput screening to coordinately investigate the effect of E5 on the expression of host protein-coding and microRNA genes. MicroRNAs form a class of 22nt long noncoding RNAs with regulatory activity. Among the altered cellular microRNAs we focus on the alteration in the expression of miR-146a, miR-203 and miR-324-5p and their target genes in a time interval of 96 hours of E5 induction. Our results indicate that HPV infection and subsequent transformation take place through complex regulatory patterns of gene expression in the host cells, part of which are regulated by the E5 protein.

## Introduction

Human papillomavirus (HPV) infection is the major cause of cervical cancer [1] and an important etiologic agent in other anogenital cancers (reviewed in [2] and [3]). Cervical infections by high-risk HPV genotypes cause virtually all cervical cancers and their immediate precursors worldwide [4]. The most prevalent HPV type found in cervical cancer is HPV 16, which encodes three oncoproteins: E5, E6 and E7. The E6 and E7 oncoproteins can bind to and stimulate the degradation of the tumor suppressors p53 [5], [6], [7] and pRb [8]. Their oncogenic potentials are largely correlated with these interactions [9], [10] but their interference with the functions of other intracellular proteins plays an important role as well [11], [12].

The E5 protein is a 83 amino acid long highly hydrophobic peptide associated with cellular membranes [13], [14], [15], [16], [17]. It has been reported to transform tissue-cultured murine fibroblasts and keratinocytes alone [18], [19] as well as to enhance the immortalization potential of E6 and E7 proteins [20]. HPV 16 E5 increases tumorigenicity in nude mice [21] and contributes to skin carcinogenesis in transgenic mice [22], [23]. It has been suggested that HPV 16 E5 acts as an oncogene primarily by enhancing the activation of the epidermal growth factor receptor in a ligand-dependent manner [19], [24], [25], [26], but the mechanisms of E5 action have not yet been established, due to a limited number of studies. Very recently it was suggested that E5 alone might have high oncogenic potential, because E5 transgenic mice were shown to develop cervical cancer after prolonged estrogen treatment [27]. Additionally, E5 potentiated the effect of E6 and E7 oncogenes in inducing cervical disease.

We have previously shown that E5 alters the expression of a number of host protein coding genes in cultured human keratinocytes [28]. Specifically, we observed that genes implicated in cell motility and cell adhesion are affected by E5 expression. We also showed enhanced motility of E5 expressing cells in an *in vivo* wound healing experiment, which suggests that E5 is implicated in the carcinogenic process [28].

MicroRNAs (miRNAs) are 20–25 nucleotides long non-coding RNAs which modulate gene expression by binding to complementary segments present in the 3′ UTR of the mRNAs of protein coding genes [29]. MicroRNAs are found in the human genome as independent loci or within intronic regions of other genes [30], [31] and they are usually transcribed by RNA polymerase II as primary miRNAs (pri-miRNAa) [32]. Pri-miRNAs are cleaved to pre-miRNAs, which are exported from the nucleus in a process involving the Exportin-5 protein. Intronic pre-miRNAs are generated as a product of splicing of the host gene [33]. In the cytoplasm, the pre-miRNA hairpins are cleaved by the RNase III enzyme Dicer [34] and the mature miRNAs are incorporated into the RNA-induced silencing complex (RISC), where they bind to their targets.

Expression of microRNAs is altered in a number of human diseases spanning from psychiatric disorders [35] to several cancers [36]. Moreover, they play a major role in regulating host gene expression in many viral infections [37]. Contrary to several other DNA tumor viruses, no miRNA species encoded by papillomaviruses have been found [38], [39], [40]. However, alterations in cellular miRNA patterns in cervical cancer tissue or cervical cancer cells have been reported [39], [40], [41]. Downregulation of human miR-218 in cervical cancer cells was specifically addressed to the HPV 16 E6 oncogene, and other high-risk HPV but not low-risk HPV E6 proteins were shown to have similar effect [41]. Human miR-218 functions by downregulating the expression of its target gene LAMB3, which is a component of the laminin-5 receptor expressed in the basal lamina of the epithelium. Laminin-5 enhances cellular migration and tumorigenicity, and its previously known overexpression in cervical cancer could thus be shown to be, at least partially, due to miR-218. The same authors showed downregulation of the tumor-suppressive miR-34a due to HPV E6 oncogene expression [41]. miR-21 has been identified as a cancer-associated miRNA overexpressed in many cancers including cervical cancer [39], [40]. It was recently shown that inhibition of miR-21 in HPV 18-containing HeLa cervical cancer cells causes a strong suppression of cell proliferation [42]. Downregulation of miR-143 in cervical cancer cell lines has also been reported [39]. It thus seems obvious that, similar to other cancers, microRNAs also play an important role in the development of cervical cancer.

Here, in order to explore the specific effects of the E5 oncogene on genome-wide expression of known human microRNAs as well as protein coding genes, we carried out DNA microarray experiments in human epithelial HaCaT cells for a time frame of 96 hours after induction of E5 expression.

Following the assumption that microRNAs are inversely expressed to their targets, regulatory loops of the differentially expressed genes were inferred.

## Materials and Methods

### Cell Cultures

HaCaT human keratinocytes stably transfected with HPV 16 E5 (HaCaT-E5) under the control of a dexamethasone inducible promoter or with the empty vector pMSG (HaCaT-pMSG) as a control [43] were used. Cells were grown in Dulbecco's modified Eagle's growth medium supplemented with 10% fetal bovine serum, glutamine, and penicillin–streptomycin to 70–80% confluence. The cells were then serum starved for 24 h and induced with 1 µM dexamethasone (Sigma-Aldrich Inc., Saint Louis, MO) for different times for analysis. Comparisons were performed between E5 and control cells treated in a similar manner.

### Analysis of mRNA expression using DNA microarrays

Total RNA was isolated from confluent cell cultures using TriPure reagent (Roche Applied Science, Indianapolis, IN) after 0, 2, 4, 24, 48,72 and 96 hour induction. RNA was quantitated in NanoDrop and the amount as well as the quality was confirmed in Agilent 2100 Bioanalyzer (Agilent Technologies, Rockville, MD). The samples (500–1000 ng) were indirectly labeled using the T7 amplification method (Amino Allyl MessageAmp™ II aRNA Amplification Kit; Ambion, Austin, TX) according to the manufacturer's instructions. aRNA (5** µ**g/sample) was labeled using monoreactive Cy3 and Cy5 dyes (GE Healthcare, Buckinghamshire, UK) or monoreactive Alexa 488 (Invitrogen, Gaithersburg, MD) followed by purification according to the manufacturer's instructions. Labeled aRNAs (800 ng/sample) were hybridized onto Agilent Whole Human Genome 4×44 K human slides according to the manufacturer's recommendations. The slides were then washed and scanned by Axon GenePix 4200 AL (Molecular Devices, Downington, PA) scanner.

### Profiling of cellular miRNA expression using DNA microarrays

E5-expressing and control cells were induced for 0, 24, 48, 72 hours in triplicates. Total RNA was isolated from confluent cell cultures (mirVana™ miRNA Isolation Kit, Ambion). RNA was quantitated in NanoDrop and the quality was confirmed by Agilent 2100 Bioanalyzer. The samples (100 ng) were labeled using Agilent miRNA labeling kit. Labeled samples were hybridized onto Agilent Human miRNA Microarray V1 slides according to the manufacturer's instructions. The slides were then washed and scanned with Axon GenePix 4200 AL scanner (Molecular Devices).

### DNA microarray analysis

Microarray data are available at the NCBI GEO database (ID GSE24908). Images from mRNA and microRNA microarrays were segmented and the median intensity of each spot was estimated by the software GenePixPro® 6.0 (Molecular Devices). The data were then imported into R software [44] and preprocessed by the BioConductor package Limma [45]. Linear model followed by moderated t-test was utilized for finding the differentially expressed genes (nominal p-value <0.001) and microRNAs (p-value <0.01 after Benjamini-Hochberg *post-hoc* correction) between E5-expressing and control cells in each time point. Additionally, analysis of variance was utilized to find expression patterns with significant alterations throughout the time points analyzed. Lists of significant genes were screened by the DAVID 6.7 annotation tools [46], [47] in order to find over-represented biological themes. Default DAVID parameters were used.

### Quantitative real-time RT-PCR

For quantitative RT-PCR, the cells were induced for 0, 2, 4, 12, 24, 36, 48, 72 and 96 h. Large RNA fraction was extracted from confluent cell cultures using mirVANA™ miRNA Isolation Kit (Ambion). Quantitative RT-PCR was performed using SYBR® Green PCR Master Mix and RT-PCR kit (Applied Biosystems, Foster City, CA) and a sequence detector ABI PRISM® 7700 (Applied Biosystems) as described previously [28].

### miRNA Taqman assays

For miRNA Taqman assays the cells were induced for 0, 4, 24, 48 and 72 h. Total RNA was isolated using the mirVana™ miRNA Isolation Kit (Ambion). Ten nanograms of total RNA were reverse transcribed using Taqman® MicroRNA Reverse Transcription Kit (Applied Biosystems). The obtained cDNA was amplified using specific Taqman ® MicroRNA assays (Applied Biosystems) for each selected miRNA in quadruplicates. The expression of β-actin mRNA from the same RNA extraction was used for normalization.

### miRNA transfections

Human Pre-miR™ miRNA Precursor for miR-203, pre-miR negative control, Anti-miR™ inhibitor for miR-146a and anti-miR negative control (Ambion) were used at a final concentration of 20 nM. To study the effects of miRNA overexpression and silencing, HaCaT-E5 and -pMSG cells (70 000/well) were reverse transfected with 20 nM miRNAs in 24-well plates using SiLentFect (Bio-Rad Laboratories, Hercules, CA), and incubated overnight. Thereafter, the cells were serum-starved for 24 h, and subsequently treated with 1 µM dexamethasone. After 48 h incubation, the cells were harvested for western blot analysis. In experiments analyzing the activation of IFN-γ or TNF-α signaling, the cells were treated with IFN-γ (10 ng/ml; Millipore, Billerica, MA) or TNF-α (20 ng/ml; Calbiochem, Merck Chemicals Ltd., Nottingham, UK) for indicated periods of time before harvesting.

### Western blotting

Subconfluent HaCaT-E5 and HaCaT-pMSG cells were serum-starved and induced for 0, 2, 4, 12, 24, 36, 48, 72 and 96 h. Total protein lysates were obtained and western blotting was performed as described previously [28]. The antibodies recognized p63 (Thermo Scientific, Fremont, CA), E-Cadherin (BD Biosciences, San Jose, CA), N-Cadherin (Zymed Laboratories, San Francisco, CA), β-Catenin (BD Biosciences, Franklin Lakes, NJ), Claudin-1 (Zymed Laboratories) and Integrin-αV (BD Biosciences). Subsequently, the membrane was incubated with secondary antibodies conjugated with fluorescent dyes: IRDye 800CW goat anti-mouse (LI-COR Biosciences, Lincoln, NE) and IRDye 680 goat anti-rabbit (LI-COR Biosciences). Protein expression was normalized against β-actin expression (Sigma Aldrich Inc.). Images were acquired with the Odyssey infrared imaging system (LI-COR Biosciences) and analyzed by the software provided by the manufacturer.

For miRNA inhibition and overexpression studies nitrocellulose membranes were first blocked in non-fat milk. Antibodies for phospho-p38, phospho-STAT1 and phospho-p42/44 (ERK1/2) as well as the antibodies for total p38, STAT1 and p42/44 were from Cell Signaling Technology Inc. (Danvers, MA,). Equal loading was confirmed by probing the same membranes for human β-actin (Sigma-Aldrich). The blots were visualized by enhanced chemiluminescence (ECL) detection system (Pierce, Thermo Scientific, Rockford, IL).

### Immunohistochemistry

Tissue samples were fixed in 10% formaline and embedded in paraffin. Collagen raft cultures were prepared using HaCaT-E5 and HaCaT-pMSG cells to produce a three-dimensional tissue culture mimicking layered epithelium, and embedded in paraffin (modified from [48]). For immunohistochemical staining, 4–5 µm sections were prepared and immunostainings were performed using the automated Ventana Discovery tissue staining instrument (Ventana Medical Systems, Tucson, AZ). Representative tissue sections from HPV-associated cervical dysplasia, normal cervical squamous epithelium and collagen raft cultures were stained using monoclonal antibodies to E-Cadherin (BD Transduction Laboratories), β-Catenin (BD Biosciences), N-Cadherin (Sigma-Aldrich Inc.), ezrin (clone 3C12 [49]) and p63 (Thermo Scientific) proteins. p16 staining (CINtec Histology Kit, mtm laboratories AG, Heidelberg, Germany) was used in staining of human tissue as a surrogate marker for HPV. Ventana DAB Map kit was used for detection, and the sections were counterstained with hematoxylin and postcounterstained with Bluing Reagent (Ventana Medical Systems). Finally, the slides were rinsed and dehydrated before mounting. The use of human tissue material was approved by the Ethical Committee of the Helsinki University Central Hospital.

### Prediction of microRNA targets

Putative targets of each miRNA were defined by combining the computational predictions of 8 popular algorithms including DIANA – microT [50], miRanda [51], miRDB [52], miRWALK [53], PicTar [54], PITA [55], RNA22 [56] and TargetScan [57]. The predicted targets were then intersected with the genes negatively correlated with their cognate miRNAs.

## Results

### mRNA and miRNA microarray analysis

The expression of protein-coding genes and microRNAs was analysed in HaCaT-E5 cells as compared to control cells after different durations of E5 induction in HaCaT cells using genome-wide microarrays (Table S1). The expression of protein-coding genes was studied 0, 2, 4, 24, 48, 72 and 96 hours after E5 induction. Alteration in gene expression was considered significant if the p-value was <0.001. The number of probes detecting differential gene expression at the different time points varied between 89 (4 h induction) and 660 (24 h induction). Sixty percent of the probes detected over-expressed transcripts at all time points in the E5-induced cells as compared to control cells, with the exception of 24 h where this rate was 45%. The gene expression differences ranged between +5.58 and −4.51 on the log2 scale (Table 1).

**Table 1:** Summary of microarray results.

TimePoint	#upreg mRNA	#downreg mRNA	#tot mRNA	#upreg miRNA	#downreg miRNA	#tot miRNA
0 h	256	176	432	0	1	1
2 h	325	191	516	NA	NA	NA
4 h	56	33	89	NA	NA	NA
24 h	296	364	660	0	2	2
48 h	336	207	543	2	1	3
72 h	179	128	307	9	7	16
96 h	453	204	657	NA	NA	NA

For each time point analyzed, the number of upregulated, downregulated, total mRNA and miRNA found differentially expressed.

Functional annotation of the lists of significantly changed genes in each time point showed peculiar representation of biological themes (Table S2). Genes involved in cell motility, cell adhesion and extracellular matrix were over-represented throughout the experiment. Similarly, several genes of the immune and inflammatory response were found significantly changed in all time points of the experiment. Interestingly, at 24 hours after HPV16-E5 induction many genes involved in cell cycle were regulated.

The effect of HPV16-E5 on the expression of host microRNAs was studied in uninduced cells as well as after 24, 48 and 72 hours from E5 induction (Table S3). Alterations in miRNA expression were considered significant if the p-value was <0.01 after Benjamini-Hochberg *post hoc* correction (Table 1).

Thirteen differentially expressed microRNAs were validated by qPCR (Table 2) and we selected miR-146a, miR-203 and miR-324_5p for further investigation based on their biological relevance. miR-146a was constantly found upregulated in E5-expressing HaCaT cells at all the time points examined. miR-324-5p was constantly downregulated in E5-expressing cells at all the time points. miR-203 remained unchanged during the first 24 hours of the experiment but was repressed at later stages (48, 72 and 96 hours).

**Table 2:** qPCR validation of miRNA microarray results.

microRNA	0 h	2 h	4 h	24 h	48 h	72 h
miR-146a	1217.8 (2.1E-16)	223.2 (4.3E-13)	1676.4 (3.1E-19)	9.98E12 (6.9E-11)	87.2 (1.9E-05)	19.8 (0.0117)
miR-203	2.3 (0.0180)	N/A	2.7 (0.0153)	2.4 (0.1135)	7.7 (0.0787)	−771.3 (0.0008)
miR-324_5p	1.2 (0.1824)	N/A	−1.4 (0.8355)	−1.3 (0.0081)	8.3 (0.0539)	−176.1 (0.0002)
miR-107	2.2 (0.0179)	N/A	7.1 (4.2E-05)	2.3 (0.1108)	7.6 (0.0364)	−64.3 (1.9E-05)
miR-106a	4.7 (5.8E-05)	N/A	7.4 (7.4E-06)	4.0 (0.0035)	34.3 (0.0182)	−77.5 (0.0612)
miR-19a	7.1 (5.6E-05)	N/A	13.2 (0.0002)	3.8 (0.0919)	69.5 (0.0016)	−289.7 (0.0012)
miR-30a_5p	3.0 (0.0066)	N/A	6.6 (0.0008)	3.7 (0.0144)	38.1 (0.0076)	−225.8 (0.0047)
miR-23b	4.9 (4.2E-05)	N/A	8.6 (5.7E-05)	5.7 (0.0001)	48.3 (0.0023)	−159.2 (0.0009)
miR-433	2.9 (0.0001)	N/A	4.0 (3.3E-05)	2.8 (0.2041)	4.5 (0.0076)	−2.2 (0.0282)
miR-539	1.9 (0.0124)	N/A	1.8 (0.0813)	−1.2 (0.0876)	10.6 (8.4E-05)	−3.1 (0.0081)
miR-624	1.9 (0.0020)	N/A	6.9 (3.3E-05)	1.7 (0.3877)	3.9 (0.0587)	−25.1 (0.0014)
miR-214	7.7 (0.0006)	N/A	7.2 (0.0012)	5.0 (0.0040)	4.4 (3.9E-05)	−1.9 (0.0255)
miR-200c	4.1 (0.0001)	N/A	9.8 (3.3E-10)	2.9 (0.1137)	17.7 (0.0153)	−47.4 (0.2241)

The miRBase id of each miRNA assayed is indicated. Additionally, the fold change in E5 as compared to control cells, and the p-value (in brackets) at each time point are reported.

### Integration of mRNA and miRNA expression

**Figure 1:**
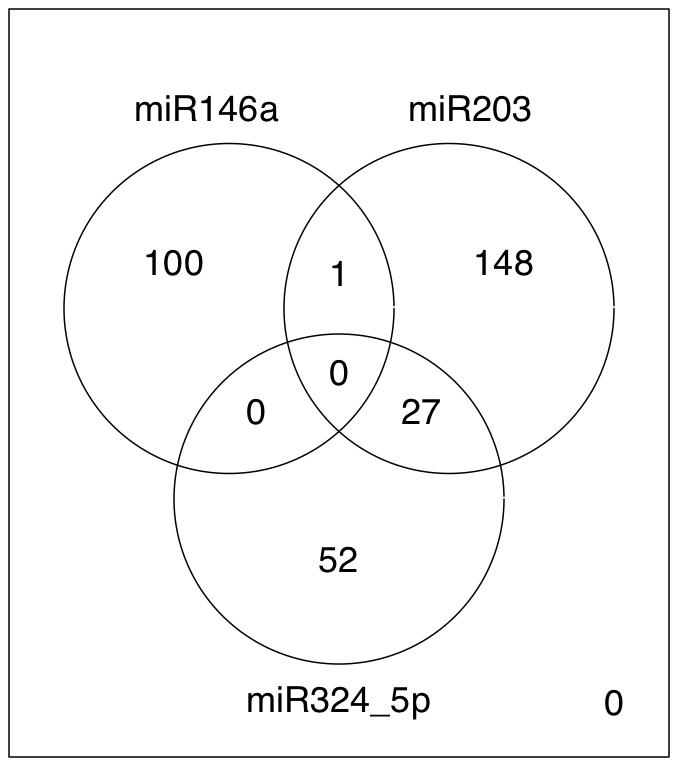
miRNA target differentially expressed genes. Venn diagram showing the number of differentially expressed genes predicted to be targets of miR-146a, miR-203 and miR-324_5p. The intersection areas indicate the number of targets shared between the miRNAs.

Integration of gene expression and microRNA expression profiles was carried out by following the assumption that the expression levels of microRNAs would be inversely correlated to the levels of their target genes. For each microRNA the differentially expressed genes showing negative correlation in DNA microarray throughout all the time points of the experiment were selected. Additionally, the putative microRNA target genes were predicted using 8 distinct algorithms, resulting in 101, 79 and 176 putative targets of miR-146a, miR-324-5p and miR-203, respectively (Figure 1, Table S4). One gene encoding a zinc finger protein, ZNF813, was predicted to be target of both miR-146a and miR-203; 27 genes were putative targets of both miR-203 and miR-324-5p; no genes were shared between miR-146a and miR-324-5p, nor by all the three microRNAs.

The miR-146a targets were enriched in genes involved in cell adhesion and cell cycle, while the biological themes represented among the miR-203 targets were associated with cell junction, cell migration and cell motility (Table S5). Genes regulating cell death and cell adhesion were predicted to be targets of the miR-324-5p.

### Validation of gene expression profiling

On the basis of the mRNA microarray results, 32 genes were selected for validation by qRT-PCR according to their biological relevance (Table 3). Additionally, ezrin expression was validated due to its relevance for cell adhesion [58]. In order to further refine the expression profiling, we measured the selected genes also at 12 and 36 hour induction, additionally to the time points investigated in microarrays. The microarray measurements were considered valid if the expression was concordant with microarray and the qRT-PCR p-value was <0.05. Altogether, 23 of 32 (72%) of the genes altered in the mRNA microarray could be confirmed. Expression of claudin-1 and integrin-β2 was upregulated. Strong upregulation of N-Cadherin (CDH2) and modest upregulation of β-Catenin (CTNNB1) was successfully verified by qRT-PCR. N-Cadherin is a predicted target of miR-324-5p. Very strong downregulation of integrin beta-like 1 and Claudin-7 was confirmed, as well as modest downregulation of tumor protein p53. Of the matrix metalloproteinases (MMPs), downregulation of MMP-12 was validated in agreement with our previous report [28]. Downregulation of PDZD2, encoding a PDZ domain protein which is a putative target of miR-146a, was validated, as well as downregulation of RACGAP1 encoding Rac GTPase activating protein 1, a putative miR-146a target. Nine genes gave discordant fold change in qRT-PCR as compared to microarray, including E-Cadherin, MMP-2 and -13. The transcript levels of several genes including Integrin-αV oscillated along with time.

**Table 3:** qPCR validation of mRNA microarray results.

GeneID	GeneSymbol	Gene Name	0 h	2 h	4 h	12 h	24 h	36 h	48 h	72 h	96 h
		HPV-16 E5	12854,6 (2,4E-07)	9657,9 (0,0002)	377,6 (1,5E-07)	20894,5 (4,4E-10)	1626,4 (2,4E-08)	91776,3 (9,9E-07)	1057700,7 (9,9E-11)	22693,6 (0,0005)	4784,5 (0,0001)
999	CDH1	E-cadherin (epithelial), cadherin 1, type 1	−2,8 (0,1091)	−3,8 (0,0457)	1,2 (0,0678)	−3,8 (0,1047)	−1,5 (0,4154)	−1,4 (8,9E-05)	−2,5 (0,0340)	−2,9 (0,0095)	−1,1 (0,8415)
1000	CDH2	N-cadherin (neuronal), cadherin 2, type 1	2,7 (0,0308)	14,0 (0,0003)	5,0 (0,0029)	5,4 (0,0018)	3,4 (0,0017)	5,3 (0,0027)	6,2 (0,0004)	4,7 (0,0068)	2,9 (0,0015)
1499	CTNNB1	catenin (cadherin-associated protein), beta 1	1,5 (0,1235)	1,8 (0,0329)	5,2 (0,0005)	2,4 (0,0042)	1,2 (0,0773)	1,9 (0,0024)	1,8 (0,0051)	1,1 (0,7189)	1,7 (0,1039)
9076	CLDN1	claudin 1	1,1 (0,7884)	3,0 (0,0090)	4,7 (0,0009)	3,6 (0,0017)	10,3 (0,0001)	18,5 (1,3E-05)	8,7 (0,0001)	4,0 (0,0017)	6,4 (0,0004)
1366	CLDN7	claudin 7	3,5 (0,0032)	−4,4 (2,8E-05)	−3,0 (0,0132)	−2,3 (0,0004)	−3,4 (0,0034)	−6,9 (0,0009)	−1,5 (0,0030)	−15,1 (0,0001)	−6,7 (0,0001)
93643	TJAP1	tight junction associated protein 1 (peripheral)	−1,4 (0,2191)	1,1 (0,6619)	4,2 (0,0014)	−3,0 (0,0012)	−2,1 (0,0402)	−1,3 (0,3457)	−3,5 (0,0026)	−3,5 (0,0194)	−2,0 (0,0024)
3685	ITGAV	integrin, alpha V (vitronectin receptor, alpha polypeptide, antigen CD51)	4,2 (0,0037)	−1,1 (0,8978)	1,3 (0,3459)	1,2 (0,7273)	−1,4 (0,8462)	−1,6 (0,2257)	1,1 (0,7298)	−2,8 (0,0585)	−1,0 (0,9653)
3689	ITGB2	integrin, beta 2 (complement component 3 receptor 3 and 4 subunit)	1,7 (0,0408)	10,4 (0,0003)	3,2 (0,0048)	6,9 (0,0007)	7,8 (0,0033)	22,0 (2,2E-05)	32,3 (8,6E-05))	33,9 (2,8E-05)	13,4 (0,0007)
9358	ITGBL1	integrin beta-like 1 (eith EGF-like repeat domains)	−2,0 (0,2186)	−12,4 (0,0017)	−3,6 (0,0462)	−3,5 (0,0171)	−6,6 (0,0069)	−7,5 (0,0047)	−7,7 (0,0028)	−20,2 (0,0002)	−7,9 (0,0004)
2697	GJA1	gap junction protein, alpha I, 43kDa	3,1 (0,0476)	3,0 (0,0358)	−2,7 (0,2671)	−1,1 (0,7336)	−4,8 (0,0031)	−4,5 (0,0465)	−1,7 (0,7707)	−10,4 (0,0015)	2,4 (0,0124)
2706	GJB2	gap junction protein, beta 2, 26 kDa	1,6 (0,6133)	2,2 (0,1820)	−10,2 (0,0118)	−4,0 (0,0101)	−6,7 (0,0040)	1,7 (0,2497)	5,7 (0,0070)	−2,1 (0,0135)	2,5 (0,0775)
4321	MMP12	matrix metalloproteinase 12	−13,5 (0,7482)	−32,5 (0,0101)	−23,6 (0,0535)	−9,5 (0,2177)	−2,9 (0,0253)	−1,7 (0,1848)	−3,8 (0,6105)	−1,3 (0,5707)	−1,4 (0,4291)
4322	MMP13	matrix metallopeptidase 13 (collagenase 3)	−6,4 (0,0002)	−14,3 (0,0005)	−4,8 (0,0020)	−2,0 (0,0017)	−2,1 (0,0119)	−8,1 (0,0012)	−14,6 (0,0005)	−3,2 (0,0101)	−3,1 (0,1887)
4313	MMP2	matrix metallopeptidase 2 (gelatinase A, 72 kDa gelatinase, 72kDa type IV collagenase)	−1,8 (0,0589)	−2,7 (0,3987)	−2,6 (0,0035)	−2,5 (0,0385)	−1,5 (0,0181)	−2,8 (0,0492)	−2,7 (0,0011)	−4,0 (0,0007)	−3,8 (0,0129)
23268	DNMBP	dynamin binding protein	−3,7 (0,0080)	−2,9 (0,0055)	−1,6 (0,2185)	−4,3 (0,0027)	−3,4 (0,0056)	−1,5 (0,3727)	−2,8 (0,0011)	−4,3 (0,0003)	−3,3 (0,0031)
146754	DNAH2	dynein, axonemal, heavy chain 2	4,6 (0,0007)	5,7 (0,0029)	12,7 (0,0003)	10,3 (0,0012)	3,1 (0,0042)	3,7 (6,9E-06)	1,3 (0,6581)	1,1 (0,7992)	3,6 (0,0015)
1767	DNAH5	dynein, axonemal, heavy chain 5	10,8 (0,0007)	3,0 (0,0010)	3,6 (0,0054)	2,6 (0,0002)	2,3 (0,0001)	3,6 (0,0015)	2,5 (2,1E-05)	3,1 (0,0015)	3,9 (0,0008)
7430	EZR	ezrin	−6,2 (0,0007)	1,4 (0,0074)	1,1 (0,5059)	−1,4 (0,1488)	−1,1 (0,7298)	−1,2 (0,3797)	−1,6 (0,0257)	−2,8 (0,0033)	−1,8 (0,1647)
2335	FN1	fibronectin 1	2,3 (0,1028)	1,7 (0,0390)	−1,2 (0,4733)	1,5 (0,2177)	1,4 (0,0456)	−2,4 (0,0002)	−3,6 (0,0104)	−2,3 (0,1344)	−5,2 (0,0072)
1307	COL16A1	collagen type 16	3,5 (0,0067)	3,2 (0,0320)	1,4 (0,4003)	10,0 (0,0010)	2,0 (0,0576)	16,5 (0,0008)	−1,2 (0,5853)	4,7 (0,0065)	13,0 (7,0E-05)
55561	CDC42BPG	CDC42 binding protein kinase gamma (DMPK-like)	−5,4 (0,0051)	−2,7 (0,0004)	2,9 (0,1503)	6,0 (0,0040)	−2,2 (0,0396)	2,2 (0,0140)	−1,8 (0,1533)	2,4 (0,1019)	2,9 (0,0060)
29127	RACGAP1	Rac GTPase activating protein 1	−8,2 (0,0045)	−5,4 (0,0358)	−3,3 (0,0092)	−3,8 (0,0175)	−2,3 (0,0129)	−2,6 (0,0002)	−5,2 (0,0006)	−9,5 (1,5E-05)	−4,7 (0,0043)
998	CDC42	cell division cycle 42 (GTP binding protein, 25kDa)	2,9 (0,0083)	−1,5 (0,6981)	−4,2 (0,0157)	−1,6 (0,2488)	−1,4 (0,1883)	1,2 (0,9216)	−2,9 (0,0302)	−4,2 (0,0348)	−1,7 (0,2156)
7157	TP53	tumor protein p53	1,3 (0,4517)	−4,5 (0,0326)	−1,4 (0,8403)	−2,8 (0,0453)	−2,7 (0,0273)	−1,9 (0,0239)	−1,6 (0,0629)	−2,9 (0,0028)	−3,5 (0,0254)
3726	JUNB	jun B proto-oncogene	1,9 (0,0211)	−2,5 (0,2629)	−18,0 (0,0052)	−3,3 (0,0026)	−2,8 (0,0299)	1,4 (0,0024)	−2,2 (0,2351)	−2,7 (0,1197)	−1,3 (0,0670)
23037	PDZD2	PDZ domain containing 2	−6,7 (0,0172)	−1,2 (0,6694)	1,5 (0,2170)	−1,6 (0,1680)	−1,0 (0,7092)	1,3 (0,7995)	−2,8 (0,0579)	−3,1 (0,0015)	−1,4 (0,5581)
4851	NOTCH1	Notch homolog 1, translocation associated (Drosophila)	−1,4 (0,0472)	6,2 (0,0007)	4,4 (0,0185)	5,9 (0,0016)	4,5 (0,0028)	9,1 (0,0025)	1,7 (0,2970)	3,8 (0,0072)	4,3 (0,0063)
7042	TGFB2	transforming growth factor beta 2	6,9 (0,0012)	1,2 (0,8566)	1,4 (0,2842)	−2,6 (0,0962)	−3,0 (0,0250)	1,6 (0,0268)	1,5 (0,4892)	1,6 (0,1547)	−1,3 (0,5478)
7127	TNFAIP2	tumor necrosis factor, alpha-induced protein 2	6,3 (5,3E-05)	3,1 (5,0E-05)	5,3 (6,0E-05)	1,9 (0,0011)	7,8 (0,0001)	3,0 (0,0110)	3,5 (0,0341)	2,9 (0,0228)	4,2 (0,0140)
664	BNIP3	BCL2/adenovirus E1B 19 kDa interacting protein 3	−14,5 (0,0005)	−18,3 (0,0001)	−14,6 (3,1E-06)	−2,4 (0,0012)	−3,4 (0,0020)	−2,4 (0,0098)	−2,1 (0,0952)	−5,2 (0,0016)	−4,2 (0,0074)
598	BCL2L1	BCL2-like 1	−1,4 (0,0748)	1,3 (0,1945)	5,2 (0,0015)	−1,3 (0,4318)	1,4 (0,2986)	1,1 (0,3976)	−2,9 (0,0031)	−5,5 (0,0053)	−1,9 (0,1357)
59	ACTA2	actin, alpha 2, smooth muscle, aorta	1,6 (0,0695)	−2,0 (0,1005)	−2,3 (0,1932)	−1,1 (0,6019)	1,9 (0,0363)	2,4 (0,1017)	−1,8 (0,1371)	−2,3 (0,1859)	−3,7 (0,0167)
3105	HLA-A	major histocompatibility complex, class I, A	−1,1 (0,7865)	−1,5 (0,0237)	1,7 (0,0098)	−1,6 (0,5345)	1,5 (0,0056)	−2,1 (0,0261)	−1,6(0,0914)	−6,3 (0,0346)	−1,1 (0,7355)

The Entrez Gene id and the name of each gene assayed are indicated. Additionally, the fold change in E5 as compared to control cells, and the p-value (in brackets) at each time point are reported.

### Protein profiling of selected genes

**Figure 2:**
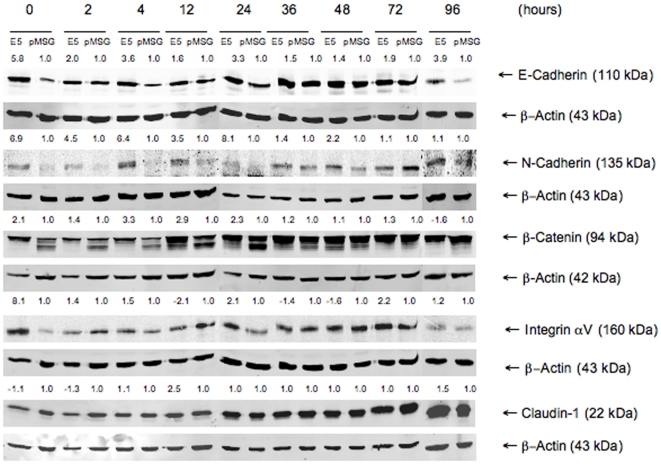
HPV E5 alters cellular protein expression. Western blots from HaCaT-E5 and -pMSG total cell lysates for E-Cadherin, N-Cadherin, β-Catenin, Integrin-αV and Claudin-1 at time points 0, 2, 4, 12, 24, 36, 48, 72 and 96 h after induction of HPV 16 E5 expression. Protein expression fold change in E5 cells as compared to control cells and normalized against β-actin is presented above each lane. Increased expression was detected for E-Cadherin, N-Cadherin and β-Catenin particularly at early time points before 24 h. Integrin-αV expression oscillated along with time and Claudin-1 was slightly downregulated at early time points.

Of the qRT-PCR validated genes, expression of Integrin-αV, Claudin-1, N-Cadherin and β-Catenin was also investigated by western blotting. Additionally, the expression of E-Cadherin was studied. Strong upregulation of N-Cadherin and modest upregulation of β-Catenin was observed in E5-expressing cells. Similarly, Integrin-αV and Claudin-1 were found to be upregulated. E-Cadherin expression was clearly upregulated in E5 cells as compared to control cells at all time points (Figure 2).

**Figure 3:**
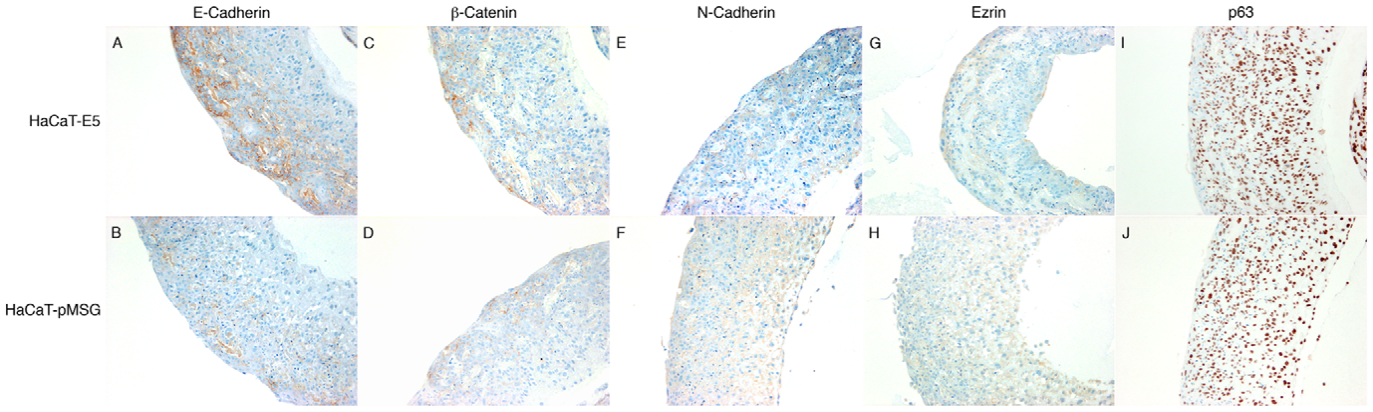
HPV 16 E5 enhances E-Cadherin expression on epithelial surface. Tissue sections from three-dimensional collagen raft cultures established from induced HaCaT-E5 cells (**A**, **C**, **E**, **G**, **I**) and HaCaT-pMSG cells (**B**, **D**, **F**, **H**, **J**) were stained for E-Cadherin (**A**, **B**), β-Catenin (**C**, **D**), N-Cadherin (**E**, **F**), Ezrin (**G**, **H**) and p63 (**I**, **J**). Surface of raft epithelium is to the left.

**Figure 4:**
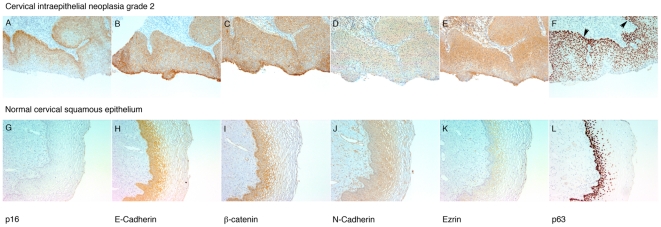
Immunohistochemical staining of a cervical intraepithelial neoplasia grade 2 (A–F) and normal cervical squamous epithelium (G–L). The expression of p16 (**A, G**), E-Cadherin (**B, H**), β-Catenin (**C, I**), N-Cadherin (**D, J**), Ezrin (**E, K**) and p63 (**F, L**) are shown. Epithelial surface is downwards in **A–F** and to the right in **G–L**. Arrowheads in F point to the basal cell layer at the bottom of squamous epithelium.

We further studied the expression of E-Cadherin, β-Catenin, N-Cadherin, ezrin and p63 proteins in three-dimensional collagen raft cultures prepared from E5 expressing and control cells (Figure 3). Membrane staining for E-Cadherin (Figure 3 A, B) and β-Catenin (C, D) was stronger towards the epithelial surface in both E5 and control raft cult1ures, and altogether the staining was stronger E5 cells, in agreement with our western blotting results. Staining for N-Cadherin (E, F) and ezrin (G, H) was equal or somewhat stronger in control cells. Also, representative human tissue samples from cervical dysplasia were stained (our own unpublished work). Figure 4 shows an example of a high-risk HPV-associated cervical intraepithelial neoplasia grade 2 case (CIN2) as well normal cervical squamous epithelium. p16 staining (Figure 4 A) was included to depict high risk HPV associated dysplasia. Normal tissue is negative for p16 (4. G). Membrane staining for E-Cadherin (B) and β-Catenin (C) was observed throughout squamous epithelium in CIN2, whereas only the bottom layers were stained in normal tissue (H, I). Most of the CIN2 epithelium stained positive for N-Cadherin (D), but more membrane staining was observed at the surface, similar to ezrin (E). The differentiated cell layers of normal squamous epithelium were not stained for N-Cadherin (J) or ezrin (K). We have previously showed colozalization of ezrin and N-Cadherin at adherens junctions in HPV 18-containing HeLa cells originating from cervical carcinoma [58].

**Figure 5:**
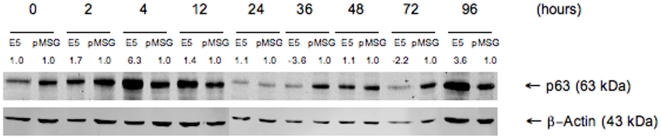
Protein expression of p63 is enhanced due to HPV E5. Western blot from HaCaT-E5 and -pMSG total cell lysates for p63 at time points 0, 2, 4, 12, 24, 36, 48, 72 and 96 h after induction of HPV 16 E5 expression. Protein expression fold change is presented above the blot when compared E5 and control in the same time point. Slight induction of p63 is detected in 4 h and 96 h time points.

In the microRNA microarray, miR-203 downregulation was observed. We therefore studied the expression of one important target of miR-203, the p63 protein, which was recently reported to be regulated by the HPV E7 protein [59]. Modest upregulation of p63 in E5 expressing cells was observed, especially in 4 h and 96 h time points (Figure 5). In three-dimensional raft cultures no clear differences were observed in p63 staining between E5-expressing and control cells (Figure 3 I, J). Staining of CIN2 tissue for p63 (Figure 4 F) decorated the nuclei in the basal and suprabasal cell layers, but towards the epithelial surface fewer nuclei were stained. The staining was clearly different from normal tissue (L), where only the bottom cells layers showed p63 staining.

### Downstream signaling effects of miR-203 overexpression and miR-146a inhibition

**Figure 6:**
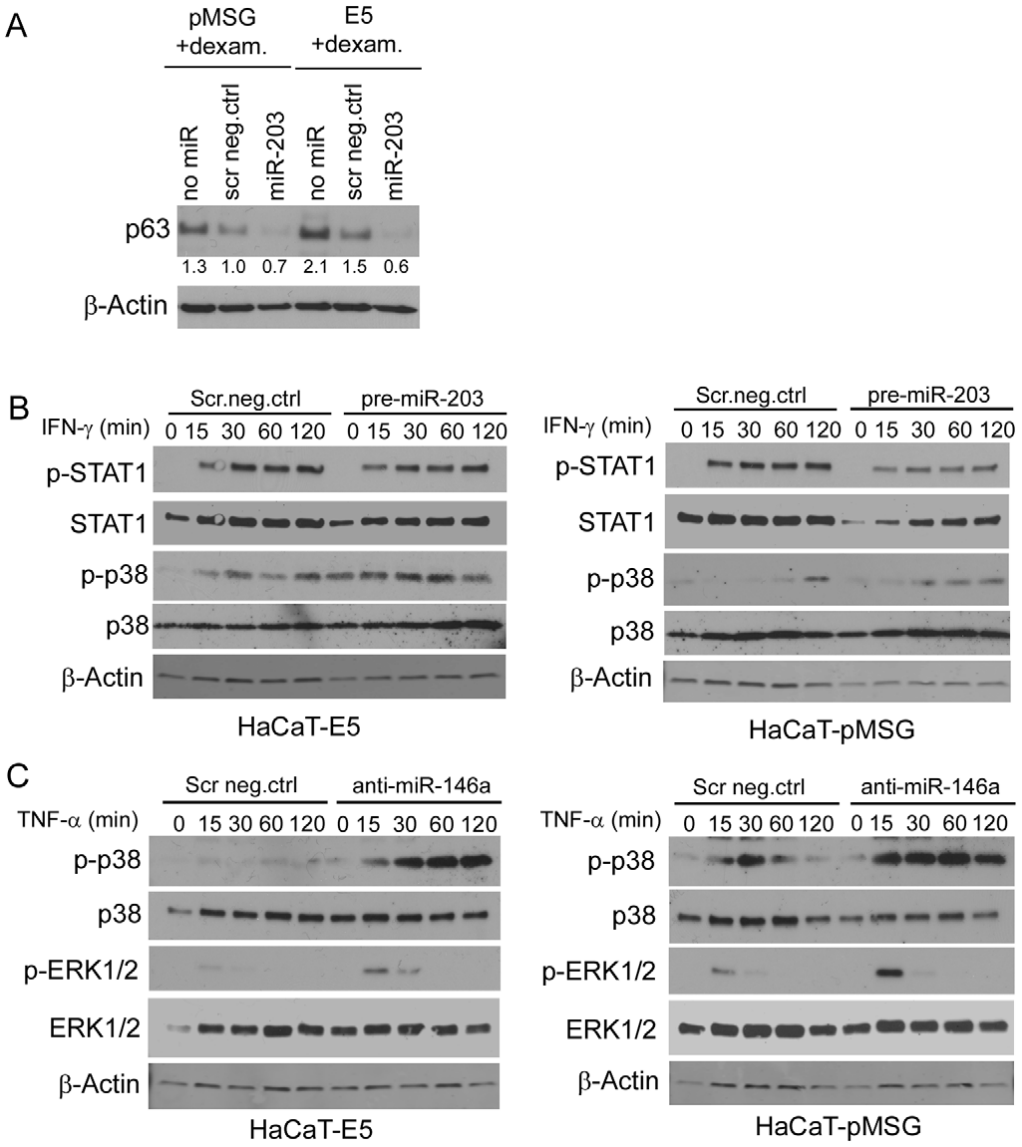
Effect of miRNA transfections on p63 expression and activation of TNF-α or IFN-γ signaling. HaCaT-E5 and –pMSG cells were transfected with 20 nM pre-miR-203 or scrambled miRNA negative control (scr neg.ctrl). After overnight incubation, the cells were serum-starved for 24 h, and subsequently treated with 1 µM dexamethasone to induce E5 expression. Forty-eight hours after induction the cells were harvested and the cell lysates analysed for p63 expression by western blotting. Equal loading was confirmed by probing the same filter with β-actin. The numbers below each lane represent p63 protein expression fold change normalized to β-actin relative to scr neg.ctrl of pMSG cells (**A**). HaCaT-E5 and -pMSG cells were transfected with scr neg.ctrl miRNA, and with either pre-miR-203 (**B**) or with anti-miR-146a (**C**). The transfection procedure was as described for **A**. Before harvesting, the cells were treated with IFN-γ (10 ng/ml) (**B**) or TNF-α (20 ng/ml) (**C**) for indicated periods of time. The cell lysates were analyzed with western blotting for phospho-p38 (p–p38), phospho-STAT1 (p-STAT1; **B**), or phospho-ERK1/2 (p-ERK1/2; **C**). The levels of total p38, STAT1, ERK1/2, and β-actin were determined as controls.

We observed a reduction of miR-203 expression in E5 cells as compared to control cells. Because p63 is a known target of miR-203 [60], we first analysed the effect of miR-203 overexpression on the levels of p63. Stronger expression of p63 was seen in E5 cells than on control cells (Figure 5, Figure 6 A), but miR-203 overexpression completely abolished the expression of p63 in both cells. Further, to evaluate the involvement of miR-203 in the regulation of inflammatory responses [61], we studied the effect of pre-miR-203 overexpression on IFN-γ signaling. Indeed, we observed increased activation (phosphorylation) of p38 in response to IFN-γ treatment, and this effect was more pronounced in E5-expressing cells (Figure 6 B). E5 cells overexpressing miR-203 have higher p-p38 levels even without IFN-γ stimulation. However, overexpression of miR-203 resulted in decreased activation of STAT1, another downstream kinase of the IFN-γ pathway. The expression of total STAT1 was somewhat decreased in miR-203 overexpressing cells as well, suggesting that additional mechanisms of STAT1 regulation by miR-203 other than IFN-γ signaling may exist.

Stronger miR-146a expression in E5 expressing cells as compared to control cells was observed. As miR-146a is known to play a role in TNF-α signaling [62], we studied whether inhibition of miR-146a by a specific anti-miRNA would affect the TNF-α-induced activation (phosphorylation) of the downstream effectors p38 and ERK1/2. In the negative control transfected cells, the levels of activated p38 (p–p38) and ERK1/2 (p-ERK1/2) were lower in E5 cells as compared to control cells (Figure 6 C, Scr.neg. ctrl). The levels of p–p38 and p-ERK1/2 in E5 cells remained undetectable after TNF-α stimulation, whereas in control cells increased activation was seen. Inhibition of miR-146a by transfection of anti-miR-146a resulted in remarkable activation of p38 and a modest activation of ERK1/2 in response to TNF-α (Figure 6 C). The response was even stronger in control cells than in E5-expressing cells.

## Discussion

In this study, the effect of HPV16 E5 oncoprotein on the expression of cellular protein-coding genes and microRNAs in HaCaT epithelial cells was investigated in genome-wide microarray experiments. Among the genes with significantly altered expression, we observed over-representation of genes involved in cell motility, cell adhesion and extracellular matrix throughout the experiment. Further, the expression of a number of genes of the immune and inflammatory response was found significantly changed in E5-expressing cells as compared to control cells in all time points of the experiment. Of interest, many genes involved in cell cycle were regulated exclusively at 24 hours after HPV16 E5 induction. Among cellular microRNAs, the altered expression of miR-146a (constantly induced by E5), miR-324-5p (constantly repressed by E5) and miR-203 (repressed at late time points) was validated and further investigated.

We observed repression of miR-203 as well as a slight induction of its target p63 in E5 expressing cells. Regulation of p63 by miR-203 was confirmed by showing that p63 was abolished upon overexpression of miR-203. miR-203 was the first identified epithelium and skin specific miRNA [61]. The p63 family of transcription factors is important in maintaining proliferation of basal epithelial cells, and the expression of p63 is diminished upon differentiation. Indeed we were able to show strong p63 expression in HPV-associated CIN, whereas in normal squamous epithelium the expression was restricted to the bottom cell layers. Although strong p63 staining in CIN is not a direct correlate of E5 expression, this finding suggests a connection between HPV oncogene expression and diminished differentiation. miR-203 has been reported to promote epithelial cell differentiation and repress ‘stemness’ of epithelial cells by repressing p63, specifically the ΔNp63 isoform [60]. miR-203 has tumor suppressor function and its downregulation has been observed in tumors [63]. Interestingly, upregulation has been reported in some cancers including colon adenocarcinoma, bladder cancer and ovarian cancer [64], [65], [66], and its overexpression in some colon or pancreatic cancers seems to correlate with patient survival [67]. Our finding suggests that E5 acts by suppressing differentiation of epithelial cells through downregulating miR-203 with subsequent upregulation of p63.

Recently it was reported that upregulation of p63 in differentiating HPV-infected cells is a consequence of the miR-203 downregulation due to E7 expression [59]. Further, it was shown that high miR-203 expression is inhibitory to HPV genome amplification [59]. This suggests that the requirement for reprogramming epithelial cells to support viral DNA amplification [68], [69] is at least partially fulfilled by the E7 protein by downregulating the expression of miR-203. Thus, the oncogenic roles of E7 as well as E5, as reported in the present study, would be mediated by miR-203.

We found strong upregulation of miR-146a throughout the time frame of 96 h in our study. This microRNA has been previously implicated in epithelial disorders such as psoriasis, and particularly in the regulation of immune responses [70]. It has been found overexpressed in breast, pancreatic, and prostate cancers [71], and underexpressed in cells derived from androgen-dependent prostate cancers [72]. miR-146a was found to be upregulated in cervical cancer tissues and to promote cell proliferation when introduced into cervical epithelial cell lines [40], suggesting that upregulation of miR-146a, among other miRNA species, plays a role in cervical carcinogenesis [40].

A number of genes of the immune and inflammatory response were found significantly changed in all time points of the microarray experiment. Human papillomaviruses are known to efficiently evade the host immune system (reviewed in [73]). HPV-associated lesions do not involve inflammation of the surrounding tissue. Cell surface MHC I and MHC II are downregulated as a function of HPV E5 and E7 oncoproteins with subsequent reduction in immune recognition [74], [75], [76], [77], [78], [79]. Nonexisting or weak immunogenicity of the E5 protein itself was suggested by the absence of antibodies to the E5 protein among HPV 16 positive cervical cancer patients (our own unpublished data), while antibodies to E7 were found in a high proportion [80]. We found strong upregulation of microRNA-146a, which is involved in negative regulation of immune responses and cytokine signaling [62], [70], [81], [82]. It may be critical in preventing excess inflammation through downregulation of IRAK1 and TRAF6, which are regulators of the TNF-α signaling pathway. miR-146 expression is induced by ligands of a subset of toll-like receptors (TLR) recognizing bacterial antigens, as well as by TNF-α and IL-1β in a NFκB-dependent manner [62]. Indeed we were able to show that TNF-α stimulation in E5 cells where miR-146a expression had been inhibited resulted in considerable activation of the downstream kinase p38 and modest activation of ERK1/2. This points out that upregulation of miR-146a by E5 may play a significant role in the attenuated immune response in HPV infections. MicroRNA-203 has been reported to target Suppressor of Cytokine Signaling-3 (SOCS-3), which is a negative regulator of IL-6 and IFN-γ signaling pathways. Suppression of SOCS-3 by miR-203 may lead to increased or elongated inflammatory responses [61]. In E5 expressing cells, downregulation of miR-203 might thus lead to enhanced expression of SOCS-3 and attenuation of the inflammatory response. In miR-203 overexpressing E5 cells we observed enhanced activation of the downstream kinase p38 in response to IFN-γ stimulation, and a similar but weaker effect was seen in control cells. This is in agreement with the role of E5 in the downregulation of immune and inflammatory responses and suggests that this effect would at least partially be mediated by miR-203. Surprisingly we observed decreased expression and activation of STAT1 after IFN-γ stimulation in both E5 and control cells overexpressing miR-203, raising the possibility of additional mechanisms of STAT1 regulation by miR-203. Indeed, by using TargetScan 5.1 [57], we found that the 3′UTR of STAT1 contains a perfect 8-mer seed match for miR-203, suggesting that STAT1 expression could be directly regulated by miR-203.

In our experiment, a much less studied species, miR-324-5p, was found constantly repressed in E5-expressing cells. It is shown to be downregulated during the early stages of colon adenocarcinogenesis in Sprague-Dawley rats [83]. Interestingly, miR-324-5p is a negative regulator of the oncogenic Hedgehog pathway in neuronal tumors, where its downregulation may contribute to tumor cell proliferation and carcinogenesis [84]. It is however, upregulated upon differentiation. Among the putative miR-324-5p targets we showed strong upregulation of N-Cadherin gene and protein expression, in agreement with downregulation of miR-324-5p. Expression of another putative target of miR-324-5p, E-Cadherin, was increased at protein level. Our data indicate that the HPV E5 oncogene may repress miR-324-5p expression in cervical epithelial cells and thus contribute to the carcinogenic process. These few data together with our findings suggest an involvement for miR-324-5p in the oncogenic functions of E5.

We previously reported alterations in the expression of cell motility and cell adhesion associated genes due to HPV 16 E5 [28]. Here we broadened the approach to comprise a time-scale analysis of cellular mRNA and microRNA expression to understand the impact of E5 in the carcinogenic process. In this study we used oligonucleotide arrays, whereas cDNA arrays were used in Kivi *et al*. [28]. In the present work we have shown upregulation of N-Cadherin and E-Cadherin proteins, as well as a slight upregulation of β-Catenin in E5 expressing cells in western blotting and also in three-dimensional collagen raft cultures. In addition to regulation by microRNAs, one possible explanation for the upregulation of E-Cadherin is increased half-life of the protein due to mechanisms involving e.g. catenins or other components of cellular junctions [85], [86]. In cervical dysplasia we showed expression of E-Cadherin, N-Cadherin and β-Catenin at cellular junctions throughout the epithelium, whereas the expression in normal tissue was restricted to the bottom layers of the epithelium. Carcinogenesis involves downregulation of E-Cadherin and disruption of E-Cadherin – β-Catenin complexes in adherens junctions, whose stability is regulated by ezrin [87], [88]. We have previously shown colocalization of ezrin in adherens junctions with N-Cadherin but no expression of E-Cadherin in HPV 18 containing HeLa cervical carcinoma cells, as well as the requirement for Rac1, phosphatidylinositol-4-phosphate 5-kinase (PIPKα) and RhoA for this localization [58]. Slight downregulation of ezrin, as observed by qPCR, might contribute to decreased cell adhesion at adherens junctions. Intriguingly, downregulation of epithelial markers such as E-Cadherin and upregulation of mesenchymal markers such as N-Cadherin is seen in epithelial-mesenchymal transition (EMT), a crucial process activated in cancer and generating cells with stem cell properties [89]. MMP-12 mRNA was also found downregulated but the protein levels remained unchanged (data not shown), confirming our earlier observation [28]. Besides its elastolytic activity, MMP-12 has broad substrate specificity for extracellular matrix components such as fibronectin, vitronectin, type IV collagen and laminin [90]. MMP-12 upregulation has been shown to promote cell proliferation in wound healing of epithelial cells [91]. Our data do not support the role of MMP-12 in carcinogenesis, and thus further studies are needed to clarify the impact of our finding.

Altogether, alterations in miRNA expression patterns due to HPV 16 E5 oncogene seem to favor increased cell proliferation and tumorigenesis and to repress epithelial differentiation. Previously reported functions of the E5 protein in downregulation of the immune response are supported by our expression microarray, as well as our miRNA microarray results regarding miR-146a, miR-203, and miR-324-5p. All of these microRNAs are also implicated in cancer. We believe that the HPV 16 E5 oncogene contributes to carcinogenesis by several mechanisms which involve regulation of cellular microRNAs and their target genes.

## Supporting Information

Table S1 **Differentially expressed mRNAs.** The file is composed of multiple sheets, for each time point analyzed. The Agilent probe id, the gene name and its description are shown. Moreover, the log fold change, the average expression, the t-test value, the p-value, the corrected p-value (after Benjamini and Hochberg post hoc correction) and the B values are also reported.
(XLS)

Table S2 **mRNA functional analysis.** The file consists of multiple sheets, for each time point analyzed. The category, the family name, the number of genes retrieved in the family, the enrichment percentage, the enrichment p-value are provided and the probe ids are also reported.
(XLS)

Table S3 **Differentially expressed microRNAs.** The Agilent probe id, the gene name and its description are shown. Moreover, the log fold change, the average expression, the t-test value, the p-value, the corrected p-value (after Benjamini and Hochberg post hoc correction) and the B values are also reported.
(XLS)

Table S4 **Differentially expressed targets of miR-146a, miR-203 and miR-324_5p.** The file consists of multiple sheets, for each microRNA analyzed. The Agilent probe id, the Entrez gene id, the miRNA correlation and the gene symbols are reported.
(XLS)

Table S5 **Functional analysis of the miRNA target genes.** The file consists of multiple sheets, for each time point analyzed. The similar functional categories are grouped in clusters with an enrichment score. The category, the family name, the number of genes retrieved in the family, the enrichment percentage, the enrichment p-value are provided and the probe ids are also reported.
(XLS)

## References

[1] Bosch FX, Manos MM, Munoz N, Sherman M, Jansen AM (1995). Prevalence of human papillomavirus in cervical cancer: a worldwide perspective. International biological study on cervical cancer (IBSCC) Study Group.. J Natl Cancer Inst.

[2] zur Hausen H (2002). Papillomaviruses and cancer: from basic studies to clinical application.. Nat Rev Cancer.

[3] Woodman CB, Collins SI, Young LS (2007). The natural history of cervical HPV infection: unresolved issues.. Nat Rev Cancer.

[4] Schiffman M, Castle PE, Jeronimo J, Rodriguez AC, Wacholder S (2007). Human papillomavirus and cervical cancer.. Lancet.

[5] Scheffner M, Werness BA, Huibregtse JM, Levine AJ, Howley PM (1990). The E6 oncoprotein encoded by human papillomavirus types 16 and 18 promotes the degradation of p53.. Cell.

[6] Scheffner M, Huibregtse JM, Vierstra RD, Howley PM (1993). The HPV-16 E6 and E6-AP complex functions as a ubiquitin-protein ligase in the ubiquitination of p53.. Cell.

[7] Werness BA, Levine AJ, Howley PM (1990). Association of human papillomavirus types 16 and 18 E6 proteins with p53.. Science.

[8] Dyson N, Howley PM, Münger K, Harlow E (1989). The human papilloma virus-16 E7 oncoprotein is able to bind to the retinoblastoma gene product.. Science.

[9] Heck DV, Yee CL, Howley PM, Münger K (1992). Efficiency of binding the retinoblastoma protein correlates with the transforming capacity of the E7 oncoproteins of the human papillomaviruses.. Proc Natl Acad Sci U S A.

[10] Nguyen M, Song S, Liem A, Androphy E, Liu Y (2002). A mutant of human papillomavirus type 16 E6 deficient in binding alpha-helix partners displays reduced oncogenic potential in vivo.. J Virol.

[11] Balsitis S, Dick F, Dyson N, Lambert PF (2006). Critical roles for non-pRb targets of human papillomavirus type 16 E7 in cervical carcinogenesis.. Cancer Res.

[12] Shai A, Brake T, Somoza C, Lambert PF (2007). The human papillomavirus E6 oncogene dysregulates the cell cycle and contributes to cervical carcinogenesis through two independent activities.. Cancer Res.

[13] Auvinen E, Alonso A, Auvinen P (2004). Human papillomavirus type 16 E5 protein colocalizes with the antiapoptotic Bcl-2 protein.. Arch Virol.

[14] Conrad M, Bubb VJ, Schlegel R (1993). The human papillomavirus type 6 and 16 E5 proteins are membrane-associated proteins which associate with the 16-kilodalton pore-forming protein.. J Virol.

[15] Lewis C, Baro MF, Marques M, Gruner M, Alonso A (2008). The first hydrophobic region of the HPV16 E5 protein determines protein cellular location and facilitates anchorage-independent growth.. Virol J.

[16] Oetke C, Auvinen E, Pawlita M, Alonso A (2000). Human papillomavirus type 16 E5 protein localizes to the Golgi apparatus but does not grossly affect cellular glycosylation.. Arch Virol.

[17] Suprynowicz FA, Disbrow GL, Krawczyk E, Simic V, Lantzky K (2008). HPV-16 E5 oncoprotein upregulates lipid raft components caveolin-1 and ganglioside GM1 at the plasma membrane of cervical cells.. Oncogene.

[18] Pim D, Collins M, Banks L (1992). Human papillomavirus type 16 E5 gene stimulates the transforming activity of the epidermal growth factor receptor.. Oncogene.

[19] Straight SW, Hinkle PM, Jewers RJ, McCance DJ (1993). The E5 oncoprotein of human papillomavirus type 16 transforms fibroblasts and effects the downregulation of the epidermal growth factor receptor in keratinocytes.. J Virol.

[20] Stöppler MC, Straight SW, Tsao G, Schlegel R, McCance DJ (1996). The E5 gene of HPV-16 enhances keratinocyte immortalization by full-length DNA.. Virology.

[21] Leechanachai P, Banks L, Moreau F, Matlashewski G (1992). The E5 gene from human papillomavirus type 16 is an oncogene which enhances growth factor-mediated signal transduction to the nucleus.. Oncogene.

[22] Genther Williams SM, Disbrow GL, Schlegel R, Lee D, Threadgill DW (2005). Requirement of epidermal growth factor receptor for hyperplasia induced by E5, a high-risk human papillomavirus oncogene.. Cancer Res.

[23] Maufort JP, Williams SM, Pitot HC, Lambert PF (2007). Human papillomavirus 16 E5 oncogene contributes to two stages of skin carcinogenesis.. Cancer Res.

[24] Crusius K, Auvinen E, Alonso A (1997). Enhancement of EGF- and PMA-mediated MAP kinase activation in cells expressing the human papillomavirus type 16 E5 protein.. Oncogene.

[25] Crusius K, Auvinen E, Steuer B, Gaissert H, Alonso A (1998). The human papillomavirus type 16 E5-protein modulates ligand-dependent activation of the EGF receptor family in the human epithelial cell line HaCaT.. Exp Cell Res.

[26] Tomakidi P, Cheng H, Kohl A, Komposch G, Alonso A (2000). Modulation of the epidermal growth factor receptor by the human papillomavirus type 16 E5 protein in raft cultures of human keratinocytes.. Eur J Cell Biol.

[27] Maufort JP, Shai A, Pitot HC, Lambert PF (2010). A role for HPV16 E5 in cervical carcinogenesis.. Cancer Res.

[28] Kivi N, Greco D, Auvinen P, Auvinen E (2008). Genes involved in cell adhesion, cell motility and mitogenic signaling are altered due to HPV 16 E5 protein expression.. Oncogene.

[29] Bartel DP (2009). MicroRNAs: target recognition and regulatory functions.. Cell.

[30] Cai X, Hagedorn CH, Cullen BR (2004). Human microRNAs are processed from capped, polyadenylated transcripts that can also function as mRNAs.. RNA.

[31] Kim YK, Kim VN (2007). Processing of intronic microRNAs.. EMBO J.

[32] Lee Y, Kim M, Han J, Yeom KH, Lee S (2004). MicroRNA genes are transcribed by RNA polymerase II.. EMBO J.

[33] Berezikov E, Chung WJ, Willis J, Cuppen E, Lai EC (2007). Mammalian mirtron genes.. Mol Cell.

[34] Murchison EP, Hannon GJ (2004). miRNAs on the move: miRNA biogenesis and the RNAi machinery.. Curr Opin Cell Biol.

[35] Maes OC, Chertkow HM, Wang E, Schipper HM (2009). MicroRNA: Implications for Alzheimer Disease and other Human CNS Disorders.. Curr Genomics.

[36] He L, Thomson JM, Hemann MT, Hernando-Monge E, Mu D (2005). A microRNA polycistron as a potential human oncogene.. Nature.

[37] Roberts AP, Jopling CL (2010). Targeting viral infection by microRNA inhibition.. Genome Biol.

[38] Cai X, Li G, Laimins LA, Cullen BR (2006). Human papillomavirus genotype 31 does not express detectable microRNA levels during latent or productive virus replication.. J Virol.

[39] Lui WO, Pourmand N, Patterson BK, Fire A (2007). Patterns of known and novel small RNAs in human cervical cancer.. Cancer Res.

[40] Wang X, Tang S, Le SY, Lu R, Rader JS (2008). Aberrant expression of oncogenic and tumor-suppressive microRNAs in cervical cancer is required for cancer cell growth.. PLoS One.

[41] Martinez I, Gardiner AS, Board KF, Monzon FA, Edwards RP (2008). Human papillomavirus type 16 reduces the expression of microRNA-218 in cervical carcinoma cells.. Oncogene.

[42] Yao Q, Xu H, Zhang QQ, Zhou H, Qu LH (2009). MicroRNA-21 promotes cell proliferation and down-regulates the expression of programmed cell death 4 (PDCD4) in HeLa cervical carcinoma cells.. Biochem Biophys Res Commun.

[43] Oelze I, Kartenbeck J, Crusius K, Alonso A (1995). Human papillomavirus type 16 E5 protein affects cell-cell communication in an epithelial cell line.. J Virol.

[44] The R Project for Statistical Computing.. http://www.r-project.org.

[45] Smyth GK (2004). Linear models and empirical bayes methods for assessing differential expression in microarray experiments.. Stat Appl Genet Mol Biol.

[46] Dennis G, Sherman BT, Hosack DA, Yang J, Gao W (2003). DAVID: Database for Annotation, Visualization, and Integrated Discovery.. Genome Biol.

[47] Huang da W, Sherman BT, Lempicki RA (2009). Systematic and integrative analysis of large gene lists using DAVID bioinformatics resources.. Nat Protoc.

[48] Lambert PF, Ozbun MA, Collins A, Holmgren S, Lee D (2005). Using an immortalized cell line to study the HPV life cycle in organotypic “raft” cultures.. Methods Mol Med.

[49] Böhling T, Turunen O, Jääskeläinen J, Carpen O, Sainio M (1996). Ezrin expression in stromal cells of capillary hemangioblastoma. An immunohistochemical survey of brain tumors.. Am J Pathol.

[50] Maragkakis M, Alexiou P, Papadopoulos GL, Reczko M, Dalamagas T (2009). Accurate microRNA target prediction correlates with protein repression levels.. BMC Bioinformatics.

[51] Betel D, Wilson M, Gabow A, Marks DS, Sander C (2008). The microRNA.org resource: targets and expression.. Nucleic Acids Res.

[52] Wang X (2008). miRDB: a microRNA target prediction and functional annotation database with a wiki interface.. RNA.

[53] Dweep H

[54] Krek A, Grün D, Poy MN, Wolf R, Rosenberg L (2005). Combinatorial microRNA target predictions.. Nat Genet.

[55] Kertesz M, Iovino N, Unnerstall U, Gaul U, Segal E (2007). The role of site accessibility in microRNA target recognition.. Nat Genet.

[56] Miranda KC, Huynh T, Tay Y, Ang YS, Tam WL (2006). A pattern-based method for the identification of MicroRNA binding sites and their corresponding heteroduplexes.. Cell.

[57] Grimson A, Farh KK, Johnston WK, Garrett-Engele P, Lim LP (2007). MicroRNA targeting specificity in mammals: determinants beyond seed pairing.. Mol Cell.

[58] Auvinen E, Kivi N, Vaheri A (2007). Regulation of ezrin localization by Rac1 and PIPK in human epithelial cells.. Exp Cell Res.

[59] Melar-New M, Laimins LA (2010). Human papillomaviruses modulate expression of microRNA 203 upon epithelial differentiation to control levels of p63 proteins.. J Virol.

[60] Lena AM, Shalom-Feuerstein R, Rivetti di Val Cervo P, Aberdam D, Knight RA (2008). miR-203 represses ‘stemness’ by repressing DeltaNp63.. Cell Death Differ.

[61] Sonkoly E, Wei T, Janson PC, Sääf A, Lundeberg L (2007). MicroRNAs: novel regulators involved in the pathogenesis of psoriasis?. PLoS One.

[62] Taganov KD, Boldin MP, Chang KJ, Baltimore D (2006). NF-kappaB-dependent induction of microRNA miR-146, an inhibitor targeted to signaling proteins of innate immune responses.. Proc Natl Acad Sci U S A.

[63] Furuta M, Kozaki KI, Tanaka S, Arii S, Imoto I (2010). miR-124 and miR-203 are epigenetically silenced tumor-suppressive microRNAs in hepatocellular carcinoma.. Carcinogenesis.

[64] Gottardo F, Liu CG, Ferracin M, Calin GA, Fassan M (2007). Micro-RNA profiling in kidney and bladder cancers.. Urol Oncol.

[65] Iorio MV, Visone R, Di Leva G, Donati V, Petrocca F (2007). MicroRNA signatures in human ovarian cancer.. Cancer Res.

[66] Schetter AJ, Leung SY, Sohn JJ, Zanetti KA, Bowman ED (2008). MicroRNA expression profiles associated with prognosis and therapeutic outcome in colon adenocarcinoma.. JAMA.

[67] Greither T, Grochola LF, Udelnow A, Lautenschläger C, Würl P (2010). Elevated expression of microRNAs 155, 203, 210 and 222 in pancreatic tumors is associated with poorer survival.. Int J Cancer.

[68] Fehrmann F, Klumpp DJ, Laimins LA (2003). Human papillomavirus type 31 E5 protein supports cell cycle progression and activates late viral functions upon epithelial differentiation.. J Virol.

[69] Genther SM, Sterling S, Duensing S, Münger K, Sattler C (2003). Quantitative role of the human papillomavirus type 16 E5 gene during the productive stage of the viral life cycle.. J Virol.

[70] Sonkoly E, Ståhle M, Pivarcsi A (2008). MicroRNAs: novel regulators in skin inflammation.. Clin Exp Dermatol.

[71] Volinia S, Calin GA, Liu CG, Ambs S, Cimmino A (2006). A microRNA expression signature of human solid tumors defines cancer gene targets.. Proc Natl Acad Sci U S A.

[72] Lin SL, Chiang A, Chang D, Ying SY (2008). Loss of mir-146a function in hormone-refractory prostate cancer.. RNA.

[73] Stanley M (2006). Immune responses to human papillomavirus.. Vaccine.

[74] Ashrafi GH, Haghshenas MR, Marchetti B, O'Brien PM, Campo MS (2005). E5 protein of human papillomavirus type 16 selectively downregulates surface HLA class I.. Int J Cancer.

[75] Ashrafi GH, Brown DR, Fife KH, Campo MS (2006). Down-regulation of MHC class I is a property common to papillomavirus E5 proteins.. Virus Res.

[76] Bottley G, Watherston OG, Hiew YL, Norrild B, Cook GP (2008). High-risk human papillomavirus E7 expression reduces cell-surface MHC class I molecules and increases susceptibility to natural killer cells.. Oncogene.

[77] Campo MS (2002). Animal models of papillomavirus pathogenesis.. Virus Res.

[78] Li W, Deng XM, Wang CX, Zhang X, Zheng GX (2010). Down-regulation of HLA class I antigen in human papillomavirus type 16 E7 expressing HaCaT cells: correlate with TAP-1 expression.. Int J Gynecol Cancer.

[79] Zhang B, Li P, Wang E, Brahmi Z, Dunn KW (2003). The E5 protein of human papillomavirus type 16 perturbs MHC class II antigen maturation in human foreskin keratinocytes treated with interferon-gamma.. Virology.

[80] Jochmus-Kudielka I, Schneider A, Braun R, Kimmig R, Koldovsky U (1989). Antibodies against the human papillomavirus type 16 early proteins in human sera: correlation of anti-E7 reactivity with cervical cancer.. J Natl Cancer Inst.

[81] Curtale G, Citarella F, Carissimi C, Goldoni M, Carucci N (2010). An emerging player in the adaptive immune response: microRNA-146a is a modulator of IL-2 expression and activation-induced cell death in T lymphocytes.. Blood.

[82] Lu LF, Boldin MP, Chaudhry A, Lin LL, Taganov KD (2010). Function of miR-146a in controlling Treg cell-mediated regulation of Th1 responses.. Cell.

[83] Davidson LA, Wang N, Shah MS, Lupton JR, Ivanov I (2009). n-3 Polyunsaturated fatty acids modulate carcinogen-directed non-coding microRNA signatures in rat colon.. Carcinogenesis.

[84] Ferretti E, De Smaele E, Miele E, Laneve P, Po A (2008). Concerted microRNA control of Hedgehog signalling in cerebellar neuronal progenitor and tumour cells.. EMBO J.

[85] Ireton RC, Davis MA, van Hengel J, Mariner DJ, Barnes K (2002). A novel role for p120 catenin in E-cadherin function.. J Cell Biol.

[86] Lozano E, Cano A (1998). Induction of mutual stabilization and retardation of tumor growth by coexpression of plakoglobin and E-cadherin in mouse skin spindle carcinoma cells.. Mol Carcinog.

[87] Hirohashi S, Kanai Y (2003). Cell adhesion system and human cancer morphogenesis.. Cancer Sci.

[88] Hiscox S, Jiang WG (1999). Ezrin regulates cell-cell and cell-matrix adhesion, a possible role with E-cadherin/beta-catenin.. J Cell Sci 112 Pt.

[89] Mani SA, Guo W, Liao MJ, Eaton EN, Ayyanan A (2008). The epithelial-mesenchymal transition generates cells with properties of stem cells.. Cell.

[90] Matsumoto S, Kobayashi T, Katoh M, Saito S, Ikeda Y (1998). Expression and localization of matrix metalloproteinase-12 in the aorta of cholesterol-fed rabbits: relationship to lesion development.. Am J Pathol.

[91] Lyu J, Joo CK (2005). Wnt-7a up-regulates matrix metalloproteinase-12 expression and promotes cell proliferation in corneal epithelial cells during wound healing.. J Biol Chem.

